# Complete Resection of Mucinous Liver Cyst Initially Masked as a Hydatid Cyst

**DOI:** 10.1155/cris/7693600

**Published:** 2025-11-14

**Authors:** Ricardo Cruzalegui, Amanda Humpire, Juan Nuñez Ju, Erick Vasquez, Cecilia Yeren

**Affiliations:** ^1^Hepatobiliary Surgery Service, Guillermo Almenara Irigoyen Hospital, Lima, Peru; ^2^Anatomo-Pathology Service, Guillermo Almenara Irigoyen Hospital, Lima, Peru

**Keywords:** cystic neoplasm, hepatic cystic mucinous, open left hepatectomy

## Abstract

**Introduction:**

Hepatic cystic mucinous neoplasm is a low-prevalence tumor with malignant potential. Due to its infrequent presentation, it is often misdiagnosed and inadequately treated. The purpose of the present work is to report a case, review the corresponding literature, determine the most optimal surgical treatment option, and contrast it with what has been performed.

**Clinical Case:**

A 53-year-old female patient with upper hemiabdomen pain and elevated serum liver enzyme levels. Computed tomography revealed a multilocular cystic liver tumor measuring 52 mm × 63 mm between segments 4 and 5. The patient underwent a first surgery, laparoscopic unroofing. The anatomopathological result was mucinous cystic neoplasm (MCN-L) without malignancy. With the result, a second surgery was scheduled to complete the resection of the remaining cyst, and an open left hepatectomy was performed.

**Discussion:**

MCN-L of the liver is an infrequent presentation and occurs in <5% of cystic liver tumors. Because this tumor has malignant potential, complete surgical resection is the best treatment option.

**Conclusion:**

We present a case of MCN-L of the liver with two-stage complete resection because this tumor, although benign, has a high potential for malignancy and recurrence.

## 1. Introduction

Hepatic cysts are the most frequent lesions of the liver, with a prevalence of approximately 20% of the population. Of these, only 5% correspond to cystic mucinous neoplasms [1]. The World Health Organization (WHO) has classified mucin-producing bile duct tumors into two distinct entities: hepatic mucinous cystic neoplasm (MCN-L) and intraductal papillary mucinous neoplasm of the bile duct (IPMN-B) [2], neoplasms that were initially called cystadenoma or cystadenocarcinoma [3].

MCN-L, first described in 1958 as a multilocular cystic lesion [4], is an infrequent presenting tumor, occurring mainly in middle-aged women [5]. It is defined as an epithelial neoplasm composed of a monolayer of mucin-producing cubic or columnar epithelium associated with ovarian-type stroma, which has no communication with the bile ducts [6]. Due to the scarce literature on this condition, misdiagnosis in the preoperative period is common; a better prognosis is obtained when surgical resection is complete (with up to 100% survival at 5 years) [3]. We present the clinical case of a patient with hepatic MCN-L treated in the hepatobiliary surgery service of our hospital.

## 2. Presentation of the Case

A 53-year-old female patient presented with an episode of abdominal pain of moderate intensity in the right upper quadrant with no previous trigger during the 3 months before hospitalization. On physical examination, the abdomen was soft, depressible, without masses, and not painful on palpation. In the laboratory, there were alterations in serum levels of liver enzymes: gamma-glutamyl transpeptidase 151 U/L (VN: 0–30), alkaline phosphatase 899 U/L (VN: 30–120 U/L), alanine transaminase 55 U/L (VN: 0–50), and aspartate transaminase 66 U/L (0–45). However, serum bilirubin level was in the normal range (0.4 mg/dL), as well as carcinoembryonic antigen (2 U/mL), CA 19.9 (2 U/mL) and alpha-fetoprotein (1.5 U/mL) values. No antibodies against Echinococcus were detected.

A previous abdominal ultrasound performed at the reference hospital describes a cystic, heterogeneous liver lesion that does not cause dilatation of the intrahepatic bile duct.

**Figure 1:**
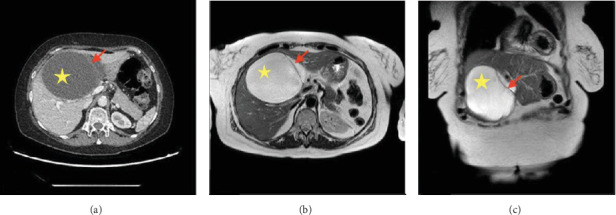
(A) Computed tomography image in axial projection shows a multilocular cystic tumor 62 mm × 53mm covering hepatic segments 4 and 5, where an internal septum is diffusely visible. (B) T2 sequence magnetic resonance in axial projection and (C) T2 sequence magnetic resonance in coronal projection shows the septum mentioned above is seen more clearly.

An abdominal CT scan revealed the presence of a 62 mm × 53 mm multilocular cystic tumor located between segments 4 and 5 of the liver (Figure 1). Magnetic resonance imaging showed the presence of a hyperintense cystic tumor in T1 with a septum and high signal intensity in the diffusion sequence, in the location already described, being considered a hepatic hydatid cyst (Figure 1). Computed tomography and magnetic resonance imaging did not reveal dilatation of the intrahepatic bile ducts or communication between the tumor and the bile ducts.

Based on these findings, a preoperative diagnosis of hepatic hydatidosis vs. hepatic cyst was made, and laparoscopic unroofing was performed, which was chosen because of the high prevalence of hydatid disease in our population [7] and the patient's epidemiological history. In our institution we opt for parenchymal-sparing surgery in benign pathology.

**Figure 2:**
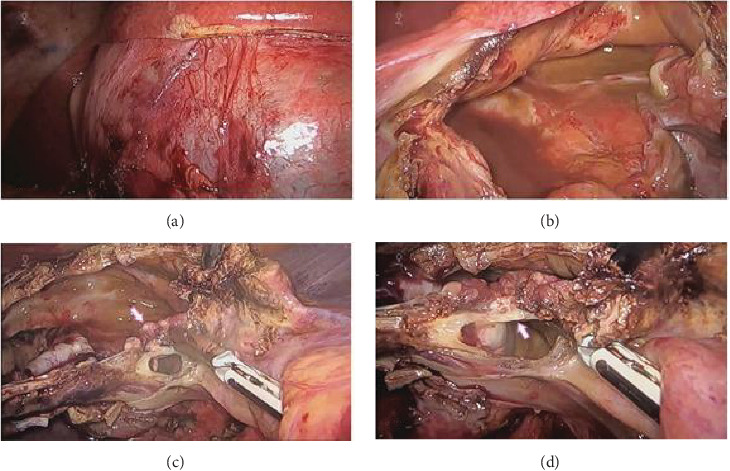
Intraoperative images corresponding to the first surgery. (A) The thickened cyst wall and (B) the cyst's internal surface can be seen. (C) The septum close to the cyst's inferior wall can be seen, (D) pointed by the white arrow.

During surgery, a cyst located between segments 4 and 5, with a thick white wall, partially protruding from the hepatic surface, with serous liquid content (Figures 2A,B) and the presence of a septum inside (Figures 2C,D); intraoperative cholangiography did not show any communication with the biliary tract. There was no spillage of the cystic content into the cavity, and intraoperative blood loss was scarce. The patient was discharged on the fourth day of surgery without postoperative complications.

**Figure 3:**
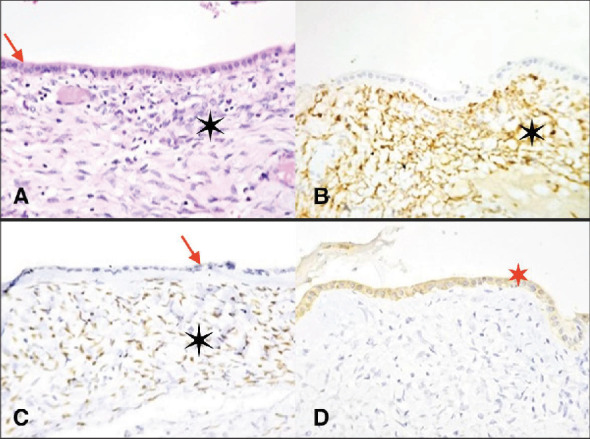
Histopathological examination: (A) Image showing a cystic formation lined by biliary-type cubic epithelium (arrow) over an ovarian-type cellular stroma (asterisk). Hematoxylin–eosin stain ×400. (B) Image showing the positivity of the stroma to CD10. Immunohistochemical staining, ×400. (C) In the image, the nuclear positivity of the stroma to estrogen is observed, but it is negative in the biliary-type epithelium. Immunohistochemical staining, ×400. (D) Image showing positivity of the biliary-type epithelium to CK7. Immunohistochemical staining, ×400.

The anatomic pathology result was reported as fibrous-looking tissue and the presence of cystic formations of serous content measuring 10 cm × 8.5 cm × 0.7 cm, with no signs of malignancy. Positive immunohistochemistry for estrogen and progesterone receptors confirmed an ovarian-type stroma. Histologically, the tumor was diagnosed as an intrahepatic mucinous cyst (Figure 3).

**Figure 4:**
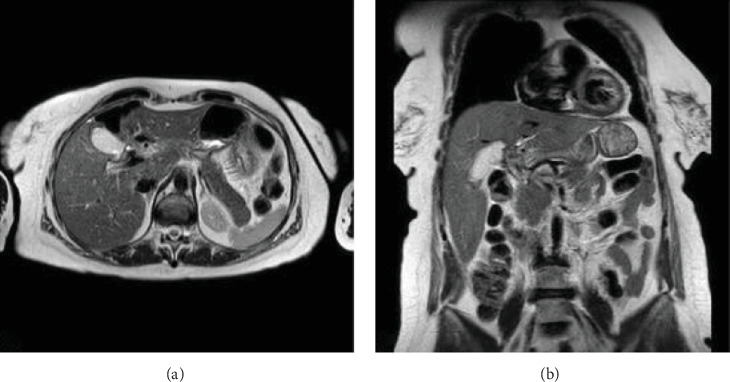
(A) T2 sequence magnetic resonance image in axial projection shows the residual wall of the cystic mucinous neoplasm, (B) T2 sequence magnetic resonance in coronal projection.

With this result, the patient was scheduled to complete the resection of the remaining mucinous cyst (Figure 4). Open left hepatectomy with lymphadenectomy was performed, where the remaining cyst wall was identified as adhered to the hepatic pedicle and enlarged lymph nodes of lymph node groups number 8 and 12. Intraoperative blood loss was 150 cc, and the patient was discharged on the fifth day of surgery without complications.

**Figure 5:**
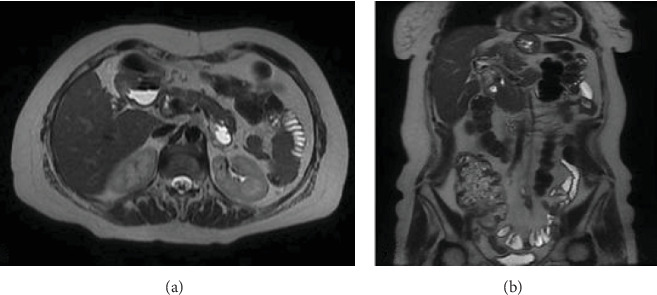
(A) T2 sequence magnetic resonance image in axial projection shows the right liver without signs of recurrence of the cystic lesion, (B) T2 sequence magnetic resonance image in coronal projection.

He is currently undergoing outpatient controls, with no signs of recurrence of the cystic lesion (Figure 5). However, he has presented variable alterations in the values of alkaline phosphatase and gamma-glutamyl transpeptidase without presenting stenosis of the main biliary tract in the control images.

## 3. Discussion

Mucinous tumors originate in organs such as the ovary or pancreas, being rare in the presentation in the liver [5], so it is scarcely reported in the literature.

MCN-L of the liver is clearly defined as an epithelial neoplasm lined with cubic or columnar cells, cyst-forming, mucin-producing, having no communication with the bile ducts, associated with an ovarian-type stroma, and expressing progesterone and estrogen receptors. It has been demonstrated that it generally occurs in women between 28 and 75 years [4]. When invasive cancer is present, the levels of tumor markers CA 19.9 and CEA in the cyst fluid are usually elevated [8].

MCN-L is known to have the potential for malignant transformation. It is classified as low, intermediate, high grade, and associated invasive carcinoma. Making a definitive diagnosis preoperatively is often difficult. In clinical practice, it should be differentiated from liver cyst, bile duct cyst, endometriosis cyst, hepatic echinococcosis, papillary bile duct tumor, and hepatic abscess, these being differential diagnoses. The main characteristic that distinguishes MCN-L is that it has no communication with the intrahepatic bile ducts, presents ovarian-type stroma, and occurs in middle-aged female patients.

In general, MCN-L presents as a low-density mass with internal septa that shows enhancement with intravenous contrast on CT. On T2-weighted MRI, MCN-L presents as a multilocular cystic mass containing fluid with varying signal intensities in the different loculi [9].

Mucinous tumors of the liver are often misdiagnosed and inadequately treated, with the possibility of disease recurrence or possible malignant transformation [10, 11]. The prognosis is good if the resection is complete [12].

Preoperative needle biopsy is challenging due to the lack of solid tissue in hepatic cystic lesions. When malignant degeneration is suspected, the most appropriate treatment is complete resection, strictly following oncologic criteria [13, 14].

In the present case, it was decided to complete the resection by performing an open left hepatectomy in a second surgical intervention after the positive histological study for mucinous neoplasm of the liver.

## 4. Conclusion

We present a case of L-CNM with complete resection in two stages because this tumor, although benign, has a high potential for malignancy and recurrence.

## Data Availability

The data that support the findings of this study are available from the corresponding author upon reasonable request.

## References

[1] Aziz H., Hamad A., Afyouni S., Kamel I. R., Pawlik T. M. (2023). Management of Mucinous Cystic Neoplasms of the Liver. *Journal of Gastrointestinal Surgery*.

[2] Nakanuma Y., Curado M.-P., Franceschi S. (2010). *WHO Classification of Tumours of the Digestive System*.

[3] Wittekind C., Fischer H. P., Ponchon T., Hamilton S. R., Aaltonen A. L. (2000). Bile Duct Cystadenoma and Cystadenocarcinoma. *WHO Classification of Tumours. Pathology and Genetics of Tumours of the Digestive System*.

[4] Edmondson H. A. (1958). *Tumors of the Liver and Intrahepatic Bile Ducts: Atlas of Tumors Pathology, Sect VII, Fascicle 25*.

[5] Zen Y., Pedica F., Patcha V. R. (2011). Mucinous Cystic Neoplasms of the Liver: A Clinicopathological Study and Comparison With Intraductal Papillary Neoplasms of the Bile Duct. *Modern Pathology*.

[6] Kubota K., Nakanuma Y., Kondo F. (2014). Clinicopathological Features and Prognosis of Mucin-Producing Bile Duct Tumor and Mucinous Cystic Tumor of the Liver: A Multi-Institutional Study by the Japan Biliary Association. *Journal of Hepato-Biliary-Pancreatic Sciences*.

[7] Vanessa G., Hernan F. *Revista Peruana de Medicina Experimental y Salud Publica*.

[8] Koffron A., Rao S., Ferrario M., Abecassis M. (2004). *Intrahepatic Biliary Cystadenoma: Role of Cyst Fluid Analysis and Surgical Management in the Laparoscopic Era*.

[9] Lee W. C. W., Tsai H. I., Lin Y. S. (2015). Intrahepatic Biliary Mucinous Cystic Neoplasms: Clinicoradiological Characteristics and Surgical results,. *BMC Gastroenterology*.

[10] Thomas K. T., Welch D., Trueblood A. (2005). Effective Treatment of Biliary Cystadenoma. *Annals of Surgery*.

[11] Oh T. H., Kim M. H., Lee S. K. (2006). Thirteen Cases of Intrahepatic Biliary Cystadenoma and Cystadenocarcinoma: A Single Center Experience. *Korean Journal of Gastroenterology*.

[12] Hansman M. F., Ryan J. A., Holmes J. H.t (2001). Management and Long-Term Follow-up of Hepatic Cysts. *American Journal of Surgery*.

[13] Suh K. S., Roh H. R., Koh Y. T., Lee K. U., Park Y. H., Kim S. W. (2000). Clinicopathologic Features of the Intraductal Growth Type of Peripheral Cholangiocarcinoma. *Hepatology*.

[14] Yang J., Wang W., Yan L. (2012). The Clinicopathological Features of Intraductal Papillary Neoplasms of the Bile Duct in a Chinese Population. *Digestive and Liver Disease*.

